# Exploring the temporal relationship between psychosocial risk factors and recurrent ischemic priapism

**DOI:** 10.1093/sexmed/qfag005

**Published:** 2026-02-16

**Authors:** Devin M Dishong, Mark Essien, Yuezhou Jing, Arthur L Burnett

**Affiliations:** Department of Urology, Johns Hopkins University School of Medicine, Baltimore, MD 21287, United States; Department of Urology, Johns Hopkins University School of Medicine, Baltimore, MD 21287, United States; Department of Urology, Johns Hopkins University School of Medicine, Baltimore, MD 21287, United States

**Keywords:** priapism, sickle cell disease, anxiety, depression, substance use

## Abstract

**Background:**

The development of recurrent ischemic priapism (RIP) is associated with mental health disorders, psychiatric medications, and substance use, but the temporal relationship between the onset of RIP and the development of these risk factors is poorly understood.

**Aim:**

This study aimed to investigate the chronology of psychosocial and substance use risk factors relative to the initial clinical presentation of RIP.

**Methods:**

We conducted a retrospective cohort study of 73 adult patients diagnosed with RIP at a single academic center, identified via manual chart review of the electronic health record. Patients presenting with RIP between 2004 and 2020 were included, with data collection extending to 2025 to ensure a minimum follow-up of nearly 5 years for all participants to minimize censoring bias and capture delayed risk factor onset.

**Outcomes:**

The date of the first RIP presentation (index event) was compared to the first documented date of several risk factors, including anxiety, depression, psychiatric medication use, and substance use.

**Results:**

In the overall cohort, there was no statistically significant difference in the proportion of any risk factor developing before versus after the onset of RIP. Further, in a sub-analysis of 35 patients with sickle cell disease (SCD), a higher proportion of patients initiated psychiatric medication after the onset of RIP, although this was not statistically significant (*P* = .0522). No other factors reached statistical significance in the SCD sub-analysis.

**Clinical implications:**

The findings suggest that RIP may be a psychologically traumatic event that contributes to the development of the same psychosocial risk factors it is associated with.

**Strengths and limitations:**

The primary strength of this study is its novel investigation into the temporal relationship between RIP and psychosocial risk factors, supported by a design that ensured a minimum follow-up period of over 4 years for all participants. Limitations are primarily related to its retrospective, single-center chart-review design.

**Conclusion:**

Our findings suggest a complex, potentially bidirectional relationship that highlights the need for integrated mental health screening for all patients with RIP, particularly for the high-risk SCD population.

## Introduction

Priapism, a persistent penile erection lasting beyond 4 h and independent of sexual stimuli, has a variety of presentations, each with significant morbidity.^1^ The most common and urgent form, ischemic priapism, results from impaired venous outflow from the corpora cavernosa and a failure of the penile detumescence mechanism, leading to a compartment syndrome of the penis that can cause irreversible corporal fibrosis and erectile dysfunction if not treated emergently.^2^ The incidence of recurrent ischemic priapism (RIP) is estimated to be from 0.34 to 0.52 per 100 000 to 1.5 per 100 000.^3^ While several risk factors are associated with the condition, sickle cell disease (SCD) is the most prominent, affecting 42%-64% of men with the disease.^4–7^ Notably, while SCD rates in the United States have remained stable, the incidence of priapism has steadily increased.^8^ This trend suggests a growing contribution from non-hematologic etiologies.

Emerging evidence points to pharmacological agents and substance use, such as alcohol, cocaine, marijuana, and tobacco, as correlates to the development of priapism.^9–13^ Additionally, psychotropic medications are a well-established cause, implicated in 15%-26% of cases.^14^ The antidepressant trazodone is one of the most frequently reported culprits, with an estimated incidence of 1 in 1000 to 1 in 10 000 users.^15–18^ Concurrently, underlying mental health conditions, including anxiety and depression, have been independently associated with priapism, potentially blurring the lines between cause and consequence.^19–21^

The psychosocial burden of RIP is substantial and can profoundly affect a patient’s quality of life. Feelings of shame and stigma may cause patients to delay seeking care, which can in turn mask serious underlying conditions.^22^ The unpredictable nature of RIP episodes, which may occur nocturnally, can lead to chronic sleep disruption and social withdrawal as patients avoid situations that might trigger an event.^23^ This lack of control over their condition, coupled with fears of permanent erectile dysfunction and potential infertility, may foster significant anxiety, hopelessness, and other mental health sequelae.^24^

Despite the body of evidence that exists for RIP, the temporal relationship between RIP risk factors and actual disease cases has yet to be elucidated. Outside of SCD, it remains unclear if conditions such as substance use, psychiatric medication use, anxiety, and depression, only predispose patients to episodes of recurrent priapism or if they develop secondarily as coping mechanisms in response to the disease burden. Therefore, in this single-center retrospective study, we sought to answer the following question: Does the onset of psychosocial and substance use risk factors temporally precede or follow the clinical presentation of RIP?

## Methods

This was a retrospective cohort study conducted at a large urban academic medical center. We utilized an electronic health record (EHR) system to identify and collect data on patients evaluated for RIP between January 1, 2004, and December 31, 2020. Data on the development of subsequent risk factors were collected for all patients through a manual chart review extending to the present (July 2025). This design ensures a minimum follow-up period of over 4 years for every patient in the cohort, providing a robust window to capture the onset of new conditions. This study was approved by our institution’s Institutional Review Board (IRB) #IRB00205900, and the requirement for individual patient consent was waived.

The study cohort was identified by searching the EHR database for the International Classification of Diseases, Tenth Revision (ICD-10) code N48.3 (Priapism). Inclusion criteria were: (1) adult patients aged 18 years or older, and (2) a confirmed diagnosis of RIP, defined as two or more documented priapism episodes. Exclusion criteria were: (1) a diagnosis of non-ischemic priapism or (2) a single, non-recurrent episode of ischemic priapism. Following the electronic query, a manual chart review was performed by study personnel to confirm eligibility and to collect data.

A standardized data collection procedure was used to extract information from the EHR via manual chart review. The index event was defined as the date of the first documented clinical presentation for an episode of RIP. Demographic data included age at presentation, race, ethnicity, and SCD status. The primary outcome was the temporal relationship between the index event and the onset of specific risk factors. Risk factors were defined as follows: a diagnosis of anxiety or depression was identified by the first documented corresponding ICD code; psychiatric medication use was defined by the date of the first prescription for an anxiolytic, antidepressant, or antipsychotic medication; substance use (marijuana, tobacco, alcohol, illicit drugs) was identified by relevant ICD codes or by explicit documentation of regular use in the social history. The date of onset for each risk factor was recorded as the first date of its documentation in the EHR. Our secondary analysis was to assess the temporal relationship between the first recurrence of RIP and the onset of specific risk factors in SCD patients only. Missing data, primarily pertinent for assessing risk factor development after RIP presentation, were due to incomplete provider documentation or gaps in longitudinal follow-up at our institution.

### Statistical analysis

One-sample binomial test was used to compare the proportions of risk factors developed before and after the index event of RIP. For the primary analysis, patients with missing data for a specific risk factor were excluded from the analysis of that variable. Statistical significance was defined as *P* < .05 for two-sided tests and all analyses were conducted in SAS 9.4 (SAS Institute Inc., Cary, NC).

## Results

A total of 73 adult patients with a confirmed diagnosis of RIP were included in this study. The median age at presentation was 31 years (IQR: 22-40). The patient cohort was predominantly African American (*n* = 48, 65.8%), followed by White (*n* = 15, 20.5%) (Table 1). Other racial identities included Other (*n* = 6, 8.2%), Unknown (*n* = 2, 2.7%), American Indian/Alaska Native (*n* = 1, 1.4%), and one patient who identified as both African American and Pacific Islander (1.4%). Regarding ethnicity, the majority of patients (*n* = 55, 75.3%) identified as Not Hispanic or Latino, while one patient (1.4%) identified as Hispanic or Latino. Ethnicity was unknown or not disclosed for the remaining 17 patients (23.3%) (Table 1).

**Table 1:** Demographic and clinical characteristics of patients with recurrent ischemic priapism. Data are presented for the overall cohort (*N* = 73). Values are shown as n (%) unless otherwise specified. Age is reported as median (IQR). IQR: Interquartile Range.

Characteristic	Overall cohort (*N* = 73)
Age at presentation, years	
Median (IQR)	31 (22-40)
Race	
African American	48 (65.8%)
White	15 (20.5%)
Other	6 (8.2%)
Unknown	2 (2.7%)
American Indian/Alaska Native	1 (1.4%)
African American and Pacific Islander	1 (1.4%)
Ethnicity	
Not Hispanic or Latino	55 (75.3%)
Unknown/not disclosed	17 (23.3%)
Hispanic or Latino	1 (1.4%)
Sickle cell disease	35 (47.9%)

The primary analysis examined the timing of diagnosis for several known risk factors relative to the first clinical presentation of RIP. For all psychosocial and substance use factors analyzed, no statistically significant difference was found between the proportion of patients who developed the risk factor before versus after the onset of RIP. The complete findings are presented in Table 2.

**Table 2:** Comparison of risk factor development before and after onset of RIP in the overall cohort. The table displays the number and percentage of patients (*N* = 73) who developed specific psychosocial factors before or after their initial RIP presentation. ^*^P-values were calculated using a one-sample binomial test to compare the proportions.

Risk factors	Before RIP *n* (%)	After RIP *n* (%)	*P*-value^^*^^
Anxiety	15 (60%)	10 (40%)	.3173
Depression	15 (56%)	12 (44%)	.5637
Psych Med Usage	16 (50%)	16 (50%)	1.0000
Marijuana	13 (52%)	12 (48%)	.8415
Tobacco	17 (59%)	12 (41%)	.3532
Alcohol	10 (42%)	14 (58%)	.4142
Illicit Drug Use	3 (43%)	4 (57%)	.7055

A sub-analysis was conducted on the subset of patients with a concomitant diagnosis of SCD (Table 3). In this group, the initiation of psychiatric medication showed a trend toward occurring more frequently after the onset of RIP. It was initiated before RIP in only three patients but after RIP in 10 patients, a finding that approached statistical significance (*P* = .0522). No other risk factors, including anxiety (*P* = .1573) or depression (*P* = .4054), reached statistical significance in this sub-analysis.

**Table 3:** Comparison of risk factor development before and after onset of RIP in patients with sickle cell disease (SCD). The table displays the number and percentage of patients in the SCD sub-analysis (*N* = 35) who developed specific risk factors before or after their initial RIP presentation. ^*^P-values were calculated using a one-sample binomial test to compare the proportions.

Risk factors	Before RIP *n* (%)	After RIP *n* (%)	*P*-value^*^
Anxiety	2 (25%)	6 (75%)	.1573
Depression	5 (38%)	8 (62%)	.4054
Psych med usage	3 (23%)	10 (77%)	.0522
Marijuana	9 (50%)	9 (50%)	1.0000
Tobacco	4 (31%)	9 (69%)	.1655
Alcohol	4 (31%)	9 (69%)	.1655
Illicit drug use	1 (33%)	2 (67%)	.5637

## Discussion

This study investigated the temporal relationship between the clinical onset of RIP and the diagnosis of several psychosocial and substance use risk factors. This work addresses a gap in the literature, as there has been minimal research on the chronology of mental health and substance abuse conditions relative to the onset of RIP. Our primary analysis revealed no statistically significant difference in the proportion of these factors developing before versus after the initial presentation of RIP. This finding suggests a complex, potentially bidirectional relationship where these conditions may act as both precursors to and consequences of the disease. A focused sub-analysis of patients with SCD, the population at highest risk for RIP, also revealed no statistically significant temporal pattern. In these patients, the initiation of psychiatric medication showed a trend towards occurring after RIP, but this did not reach statistical significance. These results provide a more nuanced understanding of the psychological impact of RIP, particularly within a medically vulnerable population.

The ambiguity in the overall cohort suggests that for many patients, the chronic pain, disruptive medical emergencies, and threat of permanent sexual dysfunction associated with RIP can precipitate significant psychological distress. The psychosocial factors that were analyzed have previously been established to be associated with priapism before its onset.^9–14^^,^^19–21^ The lack of a clear temporal divide suggests that RIP may not only arise in light of these psychosocial predispositions but also contribute to them, potentially creating a complex cycle. For individuals already managing the immense lifelong burden of a chronic, painful hematological disorder like SCD, the addition of RIP may function as a “tipping point.” It represents a distinct and severe medical trauma powerful enough to trigger a new, clinically recognized psychological crisis. The trend of increased initiation of psychiatric medication may indicate an objective marker of this distress. The experience of SCD patients with RIP is unique because, unlike patients with medication-induced priapism who can discontinue the offending agent, patients with SCD cannot remove their underlying biological predisposition. The uncertainty of a looming priapism episode and heightened risk of priapism in the SCD population, even with close medication and treatment adherence, can lead to mental exhaustion and resultant mood disorders. To cope with the effects of RIP on mental health, SCD patients may be more likely to seek out pharmacologic intervention, as supported by our observation of a statistically nonsignificant trend of increased psychiatric medication use in SCD patients after RIP presentation.

Our findings are consistent with, and expand upon, the existing literature regarding the high psychosocial burden in patients with RIP. Specifically, the number of anxiety or depression diagnoses prior to the onset of RIP in this cohort is similar to the near 50% prevalence of mood disorders observed in previous work examining recurrent priapism patients.^19^ While prior research has established this high prevalence, our study contributes a novel temporal dimension. The finding that a nearly equal number of patients develop these conditions after the diagnosis of RIP suggests that the disease itself is a significant contributor to this psychological burden, rather than mood disorders solely acting as a predisposing factor. This reinforces the interpretation of RIP as a major life event with profound and lasting psychological sequelae.

These findings have critical implications for clinical practice. Previous research has validated instruments to assess the burden on RIP through patient-reported quality of life metrics.^25^ These instruments have been used to show that priapism negatively affects psychological, sexual, and physical wellbeing.^24^ This work builds on previous research by moving beyond patient-reported metrics and utilizing objectively reported clinical data. The results from the main cohort underscore the necessity for longitudinal mental health screening for all RIP patients, as psychological comorbidities are just as likely to arise after initial disease presentation as they are to be pre-existing risk factors. Mental health disorders have been shown to be a component of psychosocial stress in patients with SCD,^26^ so this work builds on this prior knowledge and shows that RIP due to SCD may further potentiate psychosocial stress for already at risk SCD patients. Therefore, RIP patients with SCD may benefit from targeted screening for mental health conditions and a proactive consultation with mental health professionals. This model moves beyond general screening to a specific intervention for a uniquely high-risk group.

### Limitations

Our study has several limitations that must be considered. First, the retrospective design inherently limits our ability to establish causal inferences and introduces biases common to this study methodology. While the study has limitations common to retrospective designs, a key strength that mitigates these is the extended follow-up period. By capturing data for nearly 5 years after the last patient was enrolled, our study minimizes censoring bias and may provide a more accurate assessment of the psychosocial sequelae incidence following a RIP diagnosis. Second, our data were drawn from a single, urban academic center, which may restrict the generalizability of our findings to patients in different healthcare settings or geographic locations.

Further limitations stem from our reliance on data from electronic health records. The precision of our temporal analysis is constrained by the inherent lag time between a patient’s true symptom onset and the date of their formal clinical diagnosis. Reliance on provider documentation also means that incomplete or improper reporting could skew our results. Additionally, our methodology prioritized objective definitions based on ICD codes and clinical documentation to identify the presence of risk factors, rather than their severity. Consequently, we did not assess disease severity markers, such as specific suicide attempts or the degree of erectile dysfunction (eg, complete vs. residual function), nor did we evaluate social factors such as relationship status, as these variables were not consistently quantifiable across the cohort.

Specific challenges also exist in interpreting the findings within our SCD sub-cohort. Our results suggest the psychosocial effects of RIP are pronounced in these patients; however, RIP is not the only sequela of SCD that may affect mental well-being, and our analysis did not control for all potential confounders. As a congenital illness, this population carries a high baseline burden of psychological distress from chronic pain and trauma that predates the onset of RIP, making it difficult to isolate the additive impact of this specific complication.^26^ Furthermore, we could not control for the neuropsychiatric effects of SCD-specific treatments, such as chronic opioid or hydroxyurea therapy, or fully disentangle the psychological impact of RIP from that of other acute vaso-occlusive pain crises.

Finally, limitations regarding our statistical analysis must be acknowledged. We employed a one-sample binomial test because our-sample size limited the feasibility of robust multivariable modeling. Consequently, this univariable approach treats risk factors in isolation and precludes adjustment for potential covariates such as age, race, SCD status, and duration of follow-up. Future studies with larger cohorts should employ regression-based frameworks, such as logistic or Cox proportional hazards models, to control for these confounders and explore potential effect modifiers in the relationship between RIP and the development of psychosocial factors.

## Conclusion

In conclusion, this study reframes psychosocial conditions not merely as associated precursors to RIP but as frequent and significant consequences of it. This relationship is complex in the general and SCD RIP population, where RIP may act as a catalyst for clinically recognized mental health sequelae. Therefore, these findings underscore the need for an integrated care model that proactively addresses the profound and predictable psychological burden of this challenging urological condition.
